# Anti-Cariogenic Effects of *S. cerevisiae* and *S. boulardii* in *S. mutans–C. albicans* Cross-Kingdom In Vitro Models

**DOI:** 10.3390/pharmaceutics16020215

**Published:** 2024-02-01

**Authors:** Dina Yousif, Yan Wu, Alexandria Azul Gonzales, Christa Mathieu, Yan Zeng, Lee Sample, Sabrina Terando, Ting Li, Jin Xiao

**Affiliations:** 1 Eastman Institute for Oral Health, University of Rochester Medical Center, Rochester, NY 14642, USA; dina_yousif@urmc.rochester.edu (D.Y.); yan_wu@urmc.rochester.edu (Y.W.); yan_zeng@urmc.rochester.edu (Y.Z.); lee_sample@urmc.rochester.edu (L.S.); ting_li@urmc.rochester.edu (T.L.); 2 Department of Stomatology, Union Hospital, Tongji Medical College, Huazhong University of Science and Technology, Wuhan 430042, China; 3 Department of Pharmacology and Physiology, School of Medicine and Dentistry, University of Rochester Medical Center, Rochester, NY 14642, USA; alexandria_gonzales@urmc.rochester.edu; 4 VCU College of Health Professions, Virginia Commonwealth University, Richmond, VA 23284, USA; mathieucj@vcu.edu; 5 School of Arts & Sciences, University of Rochester, Rochester, NY 14627, USA; sterando@u.rochester.edu; 6 Key Laboratory of Laboratory Medical Diagnostics, Ministry of Education, Chongqing Medical University, Chongqing 400016, China

**Keywords:** *Saccharomyces cerevisiae*, *Saccharomyces boulardii*, *Candida albicans*, cross-kingdom interaction, pH, dental caries

## Abstract

Despite the well-documented health benefits of the probiotic *Saccharomyces*, its application in oral health has not been comprehensively assessed. Dental caries is a transmissible disease initiated by acid production of cariogenic bacteria and yeast, such as *Streptococcus mutans* and *Candida albicans*, on tooth enamel and followed by subsequent enamel demineralization. Here, we investigated the effect of two *Saccharomyces* strains (*Saccharomyces boulardii* and *Saccharomyces cerevisiae*) on *S. mutans*–*C. albicans* cross-kingdom interactions using a cariogenic planktonic model. Viable cells, pH changes, and gene expression were measured. *S. cerevisiae* and *S. boulardii* inhibited the growth of *C. albicans* in dual- and multi-species conditions at 4, 6, and 20 h. *Saccharomyces* also inhibited *C. albicans* hyphal formation. Furthermore, *Saccharomyces* reduced the acidity of the culture medium, which usually plummeted below pH 5 when *S. mutans* and *C. albicans* were present in the model. The presence of *Saccharomyces* maintained the culture medium above 6 even after overnight incubation, demonstrating a protective potential against dental enamel demineralization. *S. boulardii* significantly down-regulated *S. mutans atpD* and *eno* gene expression. Overall, our results shed light on a new promising candidate, *Saccharomyces*, for dental caries prevention due to its potential to create a less cariogenic environment marked by a neutral pH and reduced growth of *C. albicans*.

## 1. Introduction

Early childhood caries (ECC) is the most common chronic childhood disease worldwide [1]. Untreated ECC has a negative impact on the oral health-related quality of life of children and their families [2,3]. Oral microorganisms are associated with ECC etiopathogenesis; for example, *Streptococcus mutans* is the well-known pathogenic bacterium responsible for dental caries due to its acidogenicity and aciduric properties [4]. Recent advances in pediatric caries research also revealed the cariogenic role of fungi in ECC [5,6,7]. Specifically, *Candida albicans* has been shown to enhance cariogenicity through its synergistic interactions with *S. mutans* in producing acid, forming biofilms, and causing more severe caries [5,6,7]. Additionally, high levels of *Candida* species have been frequently reported in children with ECC [8,9,10].

Conventional measures, including oral hygiene management and pharmaceutical interventions, have been adopted for ECC prevention and treatment [3,11,12,13]. However, children remain at high risk for recurrent caries due to either low adherence to positive oral hygiene habits or the ineffectiveness of antimicrobial applications [14,15,16]. Moreover, a high relapse rate of oral candidiasis occurs among individuals wearing dentures, likely due to the conducive environment for *Candida* colonization created by the denture’s surface and the microclimate between the denture and oral mucosa [17,18]. Intriguingly, an increased adherence of both *C. albicans* and *S. mutans* to the denture acrylic base has been observed [19]. In addition to conventional antimicrobial approaches, alternative treatments, such as probiotics, have been investigated for their effects on oral health.

Probiotics are non-pathogenic live microorganisms that, when administered in appropriate quantities, can be beneficial to the health of the host [20]. Studies have shown beneficial effects of probiotic microorganisms in the oral cavity due to their inhibiting the abundance of pathogens [20]. For example, our previous work demonstrated the ability of *Lactobacillus plantarum* 14917 to inhibit the growth of *S. mutans* and *C. albicans* and cariogenic biofilm formation [21,22]. These studies elicited the potential of probiotics to inhibit cariogenic polymicrobial interactions and prevent ECC. However, the inhibitory effect of *L. plantarum* on *S. mutans* and *C. albicans* was dependent on a higher dosage of *L. plantarum* that poses challenges to clinical application [21]. Thus, given this background, it is worth exploring the potential of other probiotics in disrupting cariogenic cross-kingdom interactions.

*Saccharomyces boulardii* (*S. boulardii*) and *Saccharomyces cerevisiae* (*S. cerevisiae*) are two closely related strains commonly used as probiotics and as reagents in the preparation of food and wine. *S. boulardii* is stable over a wide range of pH levels, temperatures, and exposures to bile salts and gastrointestinal enzymes [23]. *S. boulardii* is also incapable of promoting antibiotic resistance, as the exchange of antibiotic-resistant genes between fungi and bacteria is unlikely [24,25]. Moreover, *S. boulardii* is absent from the natural gut microbiota but has been extensively studied in several gastrointestinal and systemic diseases. For example, studies have shown evidence that *S. boulardii* can prevent antibiotic-associated diarrhea [26] and prevent *Clostridium difficile*-associated colitis and traveler’s diarrhea [27,28]. *S. boulardii* has also demonstrated effectiveness in treating urinary tract and vaginal yeast infections, high cholesterol levels, lactose intolerance, teenage acne, and fever blisters [29,30,31].

Regarding oral health, two randomized controlled clinical studies [32,33] provide supporting evidence of using locally delivered probiotic *S. boulardii* as an adjunct to mechanical therapy that is used to manage periodontal disease. Moreover, Deshmukh et al. [34] assessed the impact of formulations with *S. boulardii* on oral health and found similar efficacy between chlorohexidine and probiotic mouthwashes in reducing dental plaque accumulation and promoting gingival health.

*S. cerevisiae*, commonly known as brewer’s yeast, is a unicellular fungus [35]. Studies have revealed the benefits of *S. cerevisiae* strains to both systemic and oral health. For example, daily supplements of *S. cerevisiae* delivered in single capsules were found to significantly reduce gastrointestinal symptoms of irritable bowel syndrome in both mice and humans [36,37]. *S. cerevisiae*-based intravaginal treatments also accelerated the clearance of *C. albicans* in mice with vaginal candidiasis [38]. Concerning oral health, administration of *S. cerevisiae* in the oral cavity has been shown to decrease *C. albicans* load and virulence in mice infected with oropharyngeal candidiasis [39]. Moreover, Premanathan et al. [40] observed a shorter recovery time from oral candidiasis in patients treated with topically applied *S. cerevisiae*.

Interestingly, *S. cerevisiae* shares several genes with *S. boulardii* that are involved in probiotic phenotypes [41]. These genes include *HSP150* and *YGP1*, which regulate responses to stress and acidic pH tolerance; *HSP26* and *SSA4*, which regulate heat responses; and *ARO9* and *ARO8*, which are involved in the biosynthesis of aromatic alcohols, such as phenylethanol and tryptophol [41,42]. These aromatic alcohols can inhibit the virulence of *C. albicans* [43]. Moreover, *S. boulardii* has been reported to secrete medium-chain fatty acids, mainly capric acid, with bioactivity against *C. albicans* hyphae and biofilm formation [44,45]. 

With the above-mentioned characteristics of *S. cerevisiae* and *S. boulardii*, these two species demonstrate the potential to influence cariogenic microorganisms. However, the effect of *S. boulardii* and *S. cerevisiae* on cariogenic *S. mutans* and *C. albicans* cross-kingdom interactions has not been assessed. Our study aims to fill this gap by examining the effect of probiotic *S. boulardii* and *S. cerevisiae* on the growth of *S. mutans* and *C. albicans* in a cariogenic planktonic model that mimics a high-caries-risk clinical condition. The study results will provide insight into the influence of *S. cerevisiae* and *S. boulardii* on cariogenic cross-kingdom microorganisms and expand preventative and treatment options for dental caries, such as oral application of yeast probiotics for ECC.

## 2. Materials and Methods 

### 2.1. Bacterial Strains and Starter Preparation

The microorganisms used in the study were *S. mutans* UA159, *C. albicans* SC5314, *S. boulardii* ATCC MYA796, and *S. cerevisiae* ATCC 204508. *C. albicans*, *S. mutans*, and *Saccharomyces* were recovered from frozen stock using YPD agar (BD Difco™, San Jose, CA, USA, 242720), blood agar (TSA with sheep blood, Thermo Scientific™, Waltham, MA, USA), and Yeast mold agar (BD Difco™, 271210), respectively. After 48 h incubation at 37 °C, 3–5 colonies of each species were inoculated into 10 mL of broth for overnight incubation (5% CO_2_, 37 °C). *C. albicans*, *S. boulardii,* and *S. cerevisiae* were cultured in YPD broth (BD Difco™, 242820); *S. mutans* was cultured in TSBYE broth (3% Tryptic Soy, 0.5% Yeast Extract Broth, BD Bacto™ 286220 and Gibco™ 212750, Thermo Scientific™, Waltham, MA, USA) with 1% glucose. The next day, 0.5 mL of the overnight starters was added to glass tubes containing fresh broth and incubated for 3–5 h until they reached the mid-exponential phase with desirable optical density. The morning starters were then ready to be used for the preparation of the planktonic model described below.

### 2.2. Planktonic Model

Interactions between *C. albicans*, *S. mutans*, and *Saccharomyces* species were first evaluated in planktonic conditions. The inoculation quantity of *C. albicans* (10^3^ CFU/mL) and *S. mutans* (10^5^ CFU/mL) was chosen to mimic a high-caries-risk condition in the clinical setting. Individuals who carry more than 10^5^ CFU/mL of *S. mutans* in saliva are considered to be at high risk for caries [46], while individuals who have more than 400 CFU/mL of *C. albicans* in saliva could be diagnosed with oral candidiasis using the laboratory standard [47]. The inoculation quantity of the two *Saccharomyces* species (10^7^ CFU/mL) is in the lower dose range of the probiotics used in commercial probiotic products (10^9^–10^10^ CFU as a single dosage).

Mono-species, dual-species, and multi-species models were used to assess the interaction between *C. albicans*, *S. mutans*, and *Saccharomyces* (either *S. boulardii* or *S. cerevisiae*). The planktonic models used in this study consisted of three types: mono-species, dual-species, and multi-species conditions. In the mono-species model, one of the following microorganisms: *C. albicans*, *S. mutans*, or *Saccharomyces* was incubated in 10 mL of TSBYE broth with 1% glucose for 20 h at 37 °C and 5% CO_2_. In the dual-species model, either *C. albicans* or *S. mutans* was co-cultured with one of the *Saccharomyces* species for 20 h under the same conditions. In the multi-species models, *C. albicans*, *S. mutans*, and one of the *Saccharomyces* species were mixed and cultivated for 20 h under the same circumstances. At 0, 2, 4, 6, and 20 h, the colony-forming unit per milliliter (CFU/mL) and culture media pH value were measured for each model.

To evaluate the inhibition of pseudohyphae and hyphae formation in *C. albicans* at selected time points, we placed a quantity of 20 µL of the culture medium on a glass slide and immediately observed it under a light microscope (Olympus BX43, 214, Tokyo, Japan) with a 100× oil objective (Olympus UPlanFL N 100×, Tokyo, Japan).

### 2.3. PCR and Real-Time Quantitative PCR (Real-Time qPCR)

PCR was performed in a thermal cycler (Applied Biosystems, Waltham, MA, USA), following the instructions provided by the manufacturer to assess the amplification of genes of interest. The primers used in this study are shown in Table S1 [48]. First, the yeast DNA of *Saccharomyces* species and *C. albicans* was collected from their respective overnight cultures (15 h). DNA extractions were performed using the MasterPure Yeast DNA Purification Kit (LGC Genomics, Berlin, Germany). The PCR was performed in a 50-volume containing 25 μL PCR Master Mix (2×) (Thermo Fisher Scientific, Bermen, Germany), 1 μL DNA template, 5 μL for each primer, and 14 μL nuclease-free water. The reaction was performed at 95 °C for 5 min, followed by 35 cycles of denaturation at 95 °C for 15 s, annealing at 55 °C for 30 s, and polymerization at 72 °C for 1 min, with one final extension cycle at 72 °C for 10 min. The product of the PCR was run on a pre-cast 2% agarose gel (E-gel^®^ Ex agarose gel from Invitrogen (Carlsbad, CA, USA) along with a DNA ladder (E-gel^®^ 1 kb plus DNA ladder, Invitrogen, Carlsbad, CA, USA). The gel was run for 10 min and then visualized under UV light, and the picture was saved for documentation.

Real-time qPCR was conducted to validate the expression of particular genes related to C. albicans and S. mutans virulence factors or viability. The primers used in this study are shown in Table S1 in Supplementary Materials. First, cellular RNAs were extracted from 4 mL mixture at 20 h, and 1–4 μg of purified RNA was converted to synthesize cDNAs with an iScript cDNA Synthesis Kit (Bio-Rad Laboratories, Inc., Hercules, CA, USA). The resultant cDNA and negative controls were quantitatively amplified using a QuantStudio™ 3 Real-Time PCR System (Thermo Fisher Scientific, Wilmington, DE, USA) and applied Biosystems™ PowerTrack™ SYBR Green Master Mix. A 20-volume PCR reaction comprised 2 μL cDNA template, 1 μL for each primer, 10 μL 2× SYBR-Green mix (SYBR-Green and Taq DNA Polymerase), and 6 μL nuclease-free water. To determine gene expression, three replicates for each round were set up, and relative gene expressions were calculated using the comparative ΔΔCt method. Unique core genes of *S. mutans* and *C. albicans*, namely, gyrA and ACT1, respectively, were utilized as housekeeping genes.

### 2.4. Statistical Analysis

To compare the live abundance of *C. albicans*, *S. mutans*, and *Saccharomyces* species in the planktonic models, the CFU/mL values were first converted into natural log values for statistical purposes. Of note, zero values were retained as zero. Normality tests were used to evaluate the data distribution of variables, including pH value, natural log-converted CFU/mL value, and 2^−ΔΔCT^ (real-time qRT-PCR value). To compare the difference between groups when data followed a normal distribution, the Student’s *t*-test for two groups and one-way ANOVA for more than two groups followed by a post hoc test were performed. Nevertheless, if data were not normally distributed, we used the Mann–Whitney U test to compare the results of the two groups and the Kruskal–Wallis test to compare the results for more than two groups. The statistical analysis was performed using SPSS Version 24 (SPSS Statistics for Windows, Version 24.0; IBM, Armonk, NY, USA) with a significance level of *p* < 0.05.

## 3. Results

### 3.1. Growth Profile of Saccharomyces Species

**Figure 1:**
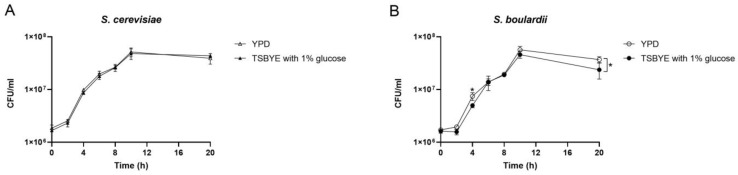
Growth curves of planktonic *Saccharomyces cerevisiae* (*S. cerevisiae*) (**A**) and *Saccharomyces boulardii* (*S. boulardii*) (**B**) in two culture mediums: YPD and TSBYE with 1% glucose. Data are presented as means (± standard deviations) of three independent experiments performed in triplicate. * Indicates a significant difference in CFU/mL between different culture media, with *p* < 0.05.

The growth curves of *S. cerevisiae* and *S. boulardii* in YPD or TSBYE with 1% glucose are shown in Figure 1. During the initial 8 h, *S. cerevisiae* grew faster than *S. boulardi*, and both reached a plateau at 10 h. *S. cerevisiae* showed similar growth curves in YPD and TSBYE with 1% glucose (Figure 1A). However, the growth of *S. boulardi* in TSBYE with 1% glucose was lower than the growth in YPD at 4 and 20 h (Figure 1B).

### 3.2. The Impact of Saccharomyces Species on C. albicans and S. mutans in the Dual-Species Conditions

**Figure 2:**
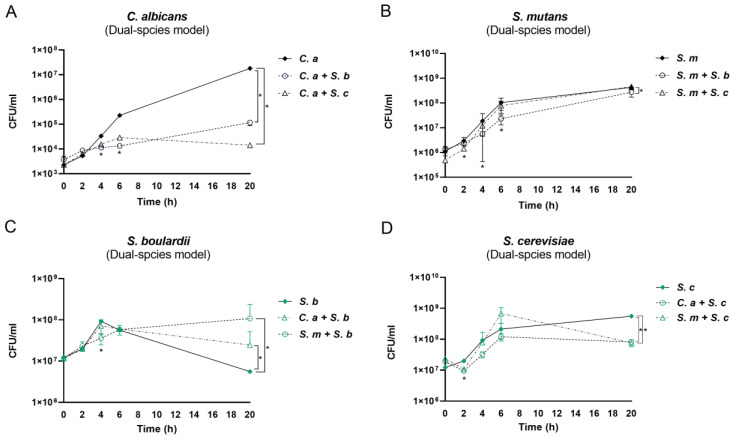
Interactions between *Saccharomyces* species and *C. albicans* or *S. mutans* in dual-species conditions. (**A**) The growth of *C. albicans* cultured with or without *Saccharomyces* species. (**B**) The growth of *S. mutans* cultured with or without *Saccharomyces* species. (**C**) The growth of *S. boulardii* cultured with or without *C. albicans/S. mutans*. (**D**) The growth of *S. cerevisiae* cultured with or without *C. albicans/S. mutans*. * Indicates a significant difference in CFU/mL between mono- and dual-species conditions, with *p* < 0.05. C. a: *C. albicans*; *S. m*: *S. mutans*; *S. c*: *S. cerevisiae*; *S. b*: *S. boulardii*.

Both *S. cerevisiae* and *S. boulardii* significantly inhibited the growth of *C. albicans* by 1 log at 4 h, 2 logs at 6 h, and 6 logs at 20 h (Figure 2A). *S. boulardii* significantly inhibited the growth of *S. mutans* at 6 and 20 h. *S. cerevisiae* significantly inhibited the growth of *S. mutans* at 2 and 4 h but failed at a later stage (Figure 2B). In contrast to the inhibited growth of *C. albicans* and *S. mutans*, *S. boulardii* grew better in the presence of *C. albicans* or *S. mutans* at 20 h (Figure 2C). However, the growth of *S. cerevisiae* in the dual-species conditions declined at 20 h (Figure 2D), which may explain the loss of its inhibitory effect on *S. mutans* at a later stage.

### 3.3. The Impact of Saccharomyces Species on C. albicans and S. mutans in the Multi-Species Conditions 

**Figure 3:**
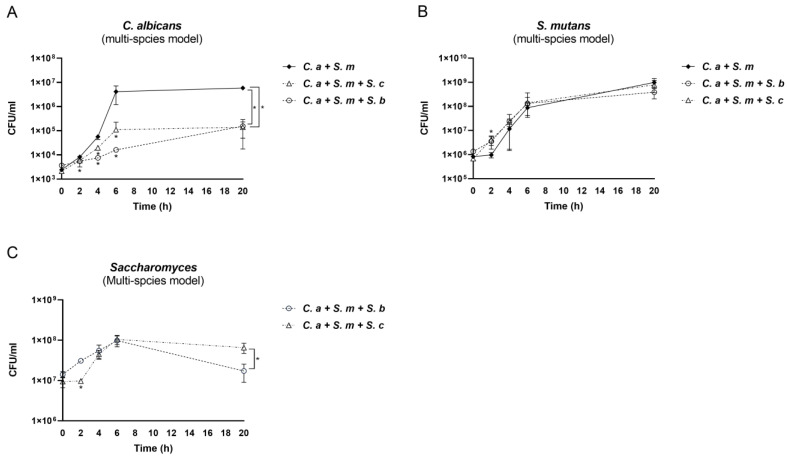
Interactions among *Saccharomyces* species, *C. albicans*, and *S. mutans* in multi-species conditions. (**A**) The growth of *C. albicans* in control (*C. a + S. m*) and *Saccharomyces* species-treated groups. (**B**) The growth of *S. mutans* in control (*C. a + S. m*) and *Saccharomyces* species-treated groups. * Indicates that *p* < 0.05 when comparing control with *Saccharomyces* species-treated groups. (**C**) The growth of *Saccharomyces* species in multi-species conditions. * Indicates that *p* < 0.05 when comparing *S. cerevisiae*- and *S. boulardii*-treated groups.

Intriguingly, both *S. cerevisiae* and *S. boulardii* significantly inhibited the growth of *C. albicans* at all time points (2, 4, 6, and 20 h) in the multi-species conditions (Figure 3A). However, *Saccharomyces* species had a dampened inhibitory effect on *S. mutans* (Figure 3B). Compared to the *C. albicans*–*S. mutans* dual-species control, *S. mutans* grew faster when together with *Saccharomyces* at the early stage; however, it grew with a reduced speed between 12 and 20 h, although the viable counts at 20 h between the groups have no statistical significance (*p* > 0.05). Between the two *Saccharomyces* species in the multi-species model, *S. cerevisiae* had a slower growth speed than *S. boulardii* at 2 h, while it had a faster growth rate at the mid and late stages (6–20 h) (Figure 3C).

### 3.4. Compositional Changes in Saccharomyces Species, C. albicans, and S. mutans in the Multi-Species Model

**Figure 4:**
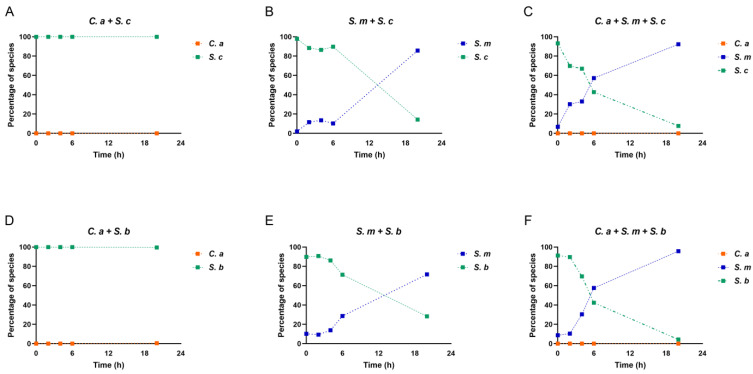
Changes in species composition in dual- and multi-species conditions. (**A**–**F**) The proportional representation of each microorganism in dual- and multi-species conditions.

Next, we assessed the compositional changes in the multi-species model over time. When *S. cerevisiae* and *C. albicans* grew together, *S. cerevisiae* showed its dominance from the beginning to the end due to the initial concentration of *S. cerevisiae* (10^7^ CFU/mL) being higher than that of *C. albicans* (10^3^ CFU/mL) (Figure 4A). When *S. cerevisiae* and *S. mutans* grew together, the initial concentration of *S. mutans* was 10^5^ CFU/mL. *S. cerevisiae* still seized its dominance from 2 h to 6 h; however, until 20 h, *S. mutans* was able to prevail after a fierce competition with *S. cerevisiae* (Figure 4B).

Next, when *S. cerevisiae* grew with *C. albicans* and *S. mutans* in the multi-species condition, a compositional switch occurred. As shown in Figure 4C, at the beginning, *S. cerevisiae* took the lead due to its highest initial concentration. Shortly, *S. mutans* displayed rapid growth rates, took over the race, and became the dominant species from 6 h to 20 h. *C. albicans*’ growth remained low over time. Intriguingly, compared to the *S. mutans*–*S. cerevisiae* dual-species condition, *S. mutans* in the multi-species model, when *C. albicans* was present, showed much stronger competitiveness against *S. cerevisiae*. This indicates an interspecies synergistic relationship between *C. albicans* and *S. mutans*, as well as synchronous antagonism between *S. cerevisiae* and *S. mutans*. A similar scenario was seen in the dual- and multi-species conditions when *S. boulardii* was present (Figure 4D–F).

### 3.5. Dynamic Changes in Culture pH in the Mono-, Dual-, and Multi-Species Conditions

**Figure 5:**
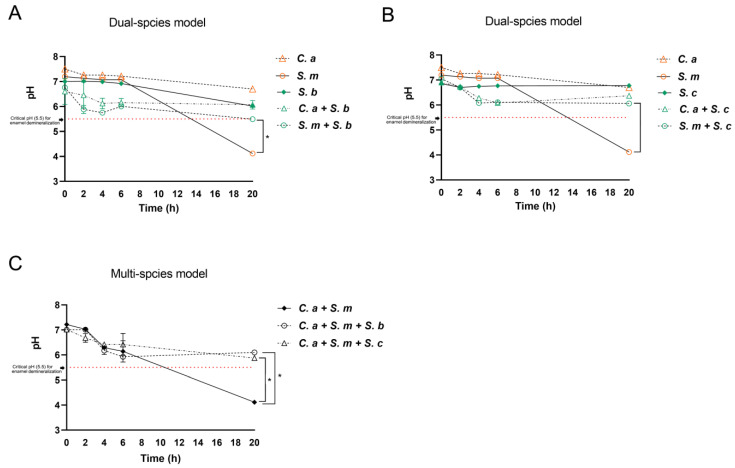
Dynamic changes in pH in the culture medium. (**A**) pH in mono-species condition and *S. boulardii* present dual-species condition. * Indicates that *p* < 0.05 when comparing dual-species (*S. b + S. m*) with mono-species (*S. m*) conditions at 20 h. (**B**) pH in mono-species condition and *S. cerevisiae* present dual-species condition. * Indicates that *p* < 0.05 when comparing dual-species (*S. c + S. m*) with mono-species (*S. m*) conditions at 20 h. (**C**) pH in control (*C. a + S. m*) and *Saccharomyces* species-treated groups. * Indicates that *p* < 0.05 when comparing *Saccharomyces* species-treated groups with control at 20 h.

Figure 5 shows the effect of *Saccharomyces* species on the environmental pH in the mono-, dual-, and multi-species models. Overall, the culture medium pH was lowered over time in all groups, particularly with a significant drop to pH 4.0 at 20 h in the *S. mutans* mono-species and *C. albicans*–*S. mutans* dual-species conditions. Significantly, the addition of either *S. cerevisiae* or *S. boulardii* neutralized the acidic environment and maintained the culture pH at 6.0 over the 20 h period, which is above the well-known critical pH of 5.5 for enamel demineralization.

### 3.6. Regulation of S. mutans and C. albicans Virulence Genes by Saccharomyces Species

To evaluate the differential gene expression between the control and the experimental conditions with added *Saccharomyces* species, qPCR was conducted at 20 h. To minimize the bias from gene expression crosstalk between *C. albicans* and *Saccharomyces species*, we first examined the expressions of *ACT1*, *EGR4*, *ECE1*, and *CHT2* in *C. albicans*, *S. cerevisiae*, or *S. boulardii*. PCR amplification products confirmed that the above genes are expressed by *C. albicans* only, not by any of the *Saccharomyces* species (Figure S1). 

**Figure 6:**
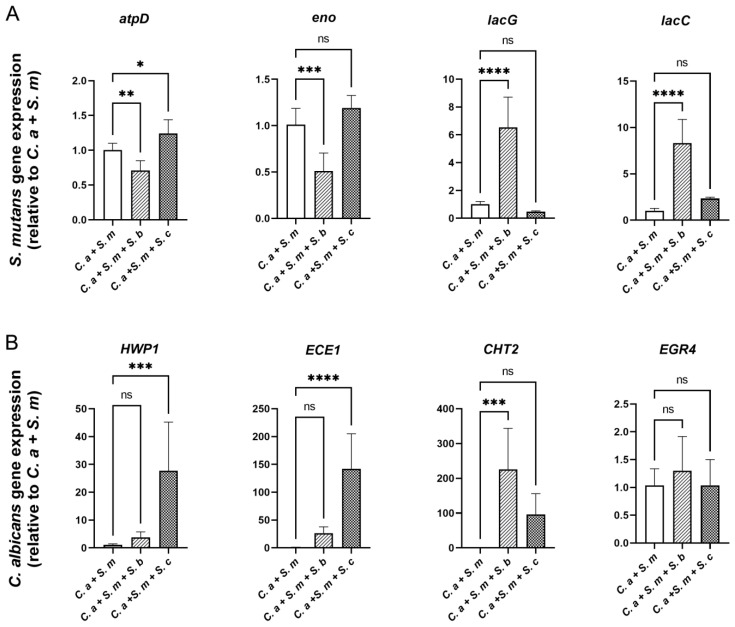
Effect of *Saccharomyces* species on the expression of *S. mutans* and *C. albicans* genes in multi-species model. qRT-PCR was performed for *S. mutans* (**A**) and *C. albicans* (**B**) genes of interest for mixed-species culture at 20 h. Relative mRNA levels were presented as ratios relative to control group (*C. a + S. m*). Results are reported as the means ± SDs of three independent experiments. *p* values were determined by one-way ANOVA with post hoc tests. * *p* < 0.05, ** *p* < 0.01, *** *p* < 0.001, **** *p* < 0.0001.

Next, as shown in Figure 6A, compared to the *C. albicans–S. mutans* dual-species control, *S. boulardii* reduced the expression of the *S. mutans* genes *atpD* (stress response gene related to ATPase complex and acid tolerance) and *eno* (associated with degradation of carbohydrates via glycolysis) by 1.4-fold (*p* < 0.01) and 2-fold (*p* < 0.001), respectively. In contrast, *lacC* and *lacG*, the genes involved in galactose metabolism, were significantly up-regulated when *S. boulardii* was added (*p* < 0.0001). The addition of *S. cerevisiae* had a negligible effect on the expression of *S. mutans* genes.

For *C. albicans* gene expression (Figure 6B), *S. cerevisiae* up-regulated the expression of *HWP1* and *ECE1*, which are associated with hyphal growth, by 27.2-fold and 74.63-fold, respectively (*p* < 0.001), whereas *S. boulardii* significantly up-regulated the expression of another *C. albicans* virulence gene, *CHT2*, which is associated with fungal wall remodeling. EGR4, however, related to antifungal medication resistance, was not statistically affected by either *S. cerevisiae* or *S. boulardii*.

### 3.7. Inhibition of C. albicans Hyphae/Pseudohyphae Formation by Saccharomyces Species

**Figure 7:**
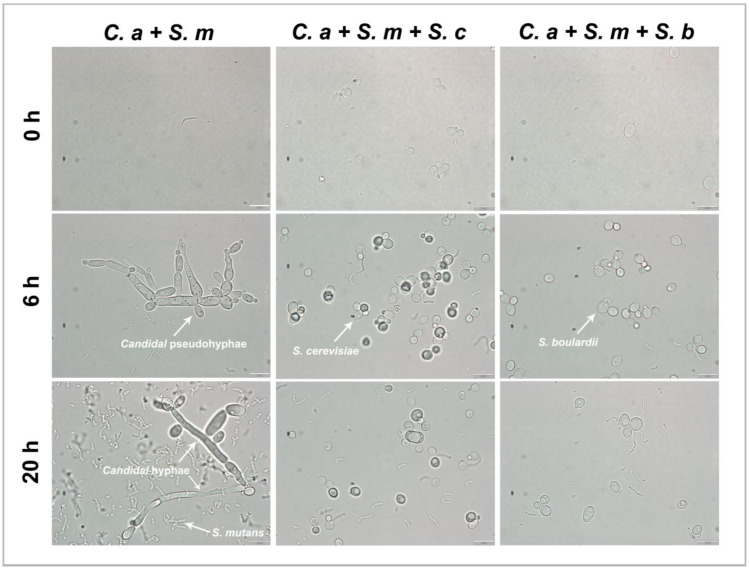
Inhibition of *Candidal* hyphae formation by *Saccharomyces* species in multi-species model at ×100 magnification. *C. albicans* that contains yeast-form, pseudohyphal, and hyphal cells can be found in control group (*C. a + S. m*) but not in *Saccharomyces* species-treated groups. These are representative images of multiple fields of view. Scale bars = 10 μm.

Inhibition of *Candida* hyphae or pseudohyphae formation was assessed by observing the culture mixture at 0 h, 6 h, and 20 h under a light microscope. In the *C. albicans–S. mutans* dual-species condition, *C. albicans* had a typical *Candidal* pseudohyphae formation at 6 h and elongated hyphal formations at 20 h. In comparison, the addition of *S. cerevisiae* or *S. boulardii* inhibited the growth of *C. albicans* in both yeast forms and the transition from yeast to hyphae or pseudohyphae form. The quantitative reduction in *C. albicans* by *S. cerevisiae* or *S. boulardii* observed in Figure 7 is consistent with the growth inhibition measured by CFUs.

## 4. Discussion

While various treatment options have been employed to control ECC, mainly by targeting cariogenic pathogens [21,22], limited studies have assessed probiotic yeast in interrupting cariogenic bacteria–fungi cross-kingdom interactions. Our study revealed novel findings that the oral health effects of *S. boulardii* and *S. cerevisiae* are not solely limited to inhibition of the growth of oral pathogens, such as *C. albicans*, but also extend to modulation of culture medium pH, influence on *C. albicans* and *S. mutans* virulence gene expression. 

Kellis et al. and Wolfe proposed a hypothesis suggesting that the fermentative capability of this yeast complex might have developed during the period when sugar-rich fruit-bearing plants became prevalent in the environment [49,50]. The sequencing of the *S. cerevisiae* genome partially supports this theory, as it uncovered substantial genetic redundancy, with a significant number of genes dedicated to sugar metabolism [51]. Today, *S. boulardii*, a probiotic yeast, is well known to interact with its host and exhibits antimicrobial activity and antitoxin and immune regulatory effects and provides various health benefits in humans [52].

In our models, the inhibitory effect of *Saccharomyces* species on the growth of *C. albicans* was notable in both the dual- and multi-species conditions. In a study conducted by Krasowska et al., it was demonstrated that introducing live *S. boulardii* cells into a *C. albicans* culture has an adverse impact on two key virulence factors of this pathogenic fungus. *S. boulardii* released factors into the medium that exhibited antagonistic effects on both the adhesion and filamentation of *C. albicans* [44]. A *S. boulardii* strain has been observed to hinder the adhesion of *C. albicans* to mucosal cell lines. Additionally, its extract diminishes cytokine-induced inflammatory responses in Caco-2 cells, evident through the suppression of IL-8 expression [53]. *S. boulardii* strains have also been identified to reduce filamentation, impede biofilm formation, and inhibit the translocation of *C. albicans* [44,54]. In our study, both *S. cerevisiae* and *S. boulardii* significantly inhibited the growth of *C. albicans* at all the time points, especially in the multi-species conditions. The competition for resources was apparent. *Saccharomyces* species competed with *C. albicans* and *S. mutans* for available nutrients. By utilizing sugars in the environment, *Saccharomyces* may limit the substrate available for the acid production by *C. albicans* and potentially reduce the metabolic activities of *C. albicans* [55]. The reduced inhibitory effect of *Saccharomyces* on the growth of *S. mutans* may be attributed to the slower growth rate of *Saccharomyces* compared to that of *S. mutans*. We also speculate that in a mixed environment, *Saccharomyces* species may demonstrate less competitiveness in utilizing available nutrients, resulting in a better proliferation of *S. mutans*.

*Saccharomyces* species, including *S. boulardii* and *S. cerevisiae*, are known for their fermentation activities. They metabolize sugars and generate organic acids (such as acetic acid and lactic acid), along with carbon dioxide and ethanol [23]. This genus can be characterized as the “sugar fungus,” particularly since its members naturally thrive in substrates rich in sweetness, such as nectar and fruits. This may explain their similar growth curves in YPD and TSBYE with 1% glucose. The optimum pH for the growth of *Saccharomyces* species is 4.5–6.5, and oxygen is important to maintain viability, but they survive under microaerophilic conditions [56]. *C. albicans* inhabits various ecological niches within the host and needs to endure a broad spectrum of environmental pH levels. The primary regulator of cytosolic pH in fungi is the plasma membrane H⁺-ATPase Pma1p. The neutral pH maintained in the culture medium when *S. boulardii* and *S. cerevisiae* interacted with *S. mutans* and *C. albicans* could be attributed to several potential mechanisms, detailed below.

Firstly, Saccharomyces may limit the substrate available for the acid production by *S. mutans* and potentially reduce the metabolic activities of *C. albicans* [55]. Saccharomyces species have been reported to produce antimicrobial compounds that could potentially inhibit the growth or metabolic activities of S. mutans and *C. albicans*, indirectly contributing to pH regulation [57]. Secondly, Saccharomyces species could also influence the expression or activity of these virulence factors, indirectly impacting the acid production and biofilm formation of S. mutans and C. albicans [44]. This is verified by our PCR results, which showed that S. boulardii significantly reduced the virulence gene expression of S. mutans (atpD and eno). The gene atpD is acid-adaptive and related to the acid stress tolerance response, while eno is related to the degradation of carbohydrates via glycolysis. Lastly, the capability of Saccharomyces species for biofilm formation and matrix production may indirectly impact the local pH environment [23].

The expressions of several genes associated with *S. mutans* virulence were altered in the multi-species models when *Saccharomyces* species were added. *S. boulardii* up-regulated two virulence genes of *S. mutans*, *lacC* and *lacG*. The tagatose 6-phosphate kinase (*lacC*) and intracellular 6-phospho-β-galactosidase (*lacG*) both participated in galactose metabolism by *S. mutans* [58]. According to Liu et al., the *S. boulardii* strain can assimilate galactose, although at a significantly lower rate compared to other *S. cerevisiae* strains [59]. This lower galactose utilization by *S. boulardii* was attributed to a single point mutation, G1278A. However, the G1278A mutation enables *S. boulardii* cells to grow on glucose [59]. When *S. boulardii* and *S. mutans* were co-cultured for carbon source utilization, to maintain energy efficiency and competitiveness, *S. boulardii* selectively utilized more rapidly metabolizable glucose, while *S. mutans* favored galactose. This may explain why *S. mutans* expressed higher levels of galactose metabolism-related genes with *S. boulardii* rather than with *S. cerevisiae*.

Finally, we found that *Saccharomyces* has a strong inhibitory effect on the crucial virulence factors of *C. albicans*, i.e., the ability to form filaments. It is a distinctive ability of *C. albicans* that it can exist in three phases, budding yeast, pseudohyphae, and hyphae [60]. *C. albicans* is recognized for adopting a filamentous morphology when exposed to serum at 37 °C, and this capability is essential for the virulence of the organism. The adaptability of the mycelial form is a crucial factor influencing drug resistance and plays a significant role during the infection stage [61]. Moreover, the transition of *C. albicans* from yeast to hyphae aids the fungi in evading macrophage phagocytosis, thereby elevating the probability of invading host tissues and causing more extensive damage [62]. In this study, hyphae/pseudohyphae formation of *C. albicans* was assessed in the *C. albicans*–*S. mutans* dual-species condition; *C. albicans* had a typical pseudohyphae formation at 6 h and elongated hyphal formations at 20 h. The addition of *S. cerevisiae* or *S. boulardii* inhibited the growth of *C. albicans* in both yeast form and the transition from yeast to hyphae or pseudohyphae form. Like our current investigation, Krasowska et al. [44] also illustrated that the suppressive impact of live *S. boulardii* cells on the filamentation of *C. albicans* strains is directly correlated with the quantity of *S. boulardii* added. Live cells of *S. boulardii* and the extract from its culture filtrate exhibited a potent inhibitory influence on the filamentation and biofilm formation of *C. albicans.* It is worth noting that despite the reduction in *C. albicans* hyphae formation by *S. cerevisiae* or *S. boulardii* observed under the microscope, the virulence gene expression of *C. albicans* (*HWP1*, *ECE1*, and *CHT2*) was found to be up-regulated by *Saccharomyces* species. This discrepancy between the gene expression and the observed hyphae formation reduction phenomena could be explained by the fact that *S. cerevisiae* or *S. boulardii* might have impacted the translation and protein synthesis process, which deserves further investigation in future studies.

Overall, our results provided evidence to fill an important gap in dental caries research by examining the effect of probiotic *S. boulardii* and *S. cerevisiae* on the growth of *S. mutans* and *C. albicans* in a cariogenic planktonic model that mimics a high-caries-risk clinical condition. The following limitations are recognized with the intriguing findings: Although our study results indicated the interactions between *Saccharomyces* species, *S. mutans* and *C. albicans*, other cariogenic factors, such as biofilm formation and enamel demineralization, need to be assessed in biofilm and animal models. Second, our study introduced glucose as the sugar challenge in the planktonic model and future studies should assess other forms of carbohydrates, such as sucrose. Third, we used qRT-PCR to assess several virulence genes; however, high-throughput methods such as RNA sequencing would offer more comprehensive understanding of the global transcriptomic changes in *S. mutans* and *C. albicans* when they interact with *Saccharomyces* species, which we plan to assess in future investigations. Fourth, different methods for probiotic application, including oral rinse, and local and topical delivery have been reported in clinical trials. Animal studies and human clinical trials are warranted to further assess the preventive and therapeutic effects of probiotic *S. boulardii* and *S. cerevisiae*.

## 5. Conclusions

To the best of our knowledge, this is the first study to demonstrate the inhibitory effect of *S. cerevisiae* and *S. boulardii* on the growth of *C. albicans* in a cariogenic planktonic model. The results shed light on a new promising candidate, *Saccharomyces*, for dental caries prevention due to its potential to create a less cariogenic environment marked by a neutral pH and reduced growth of common cariogenic pathogens.

## Data Availability

All data generated or analyzed during this study are included in this article. Further inquiries can be directed to the corresponding author.

## Supplementary Materials

The following supporting information can be downloaded at: https://www.mdpi.com/article/10.3390/pharmaceutics16020215/s1. Table S1: Primers used in PCR; Figure S1: PCR amplification products obtained with four types of primers.
