# Supplementary material for: Anti-Cariogenic Effects of S. cerevisiae and S. boulardii in S. mutans–C. albicans Cross-Kingdom In Vitro Models
Source: Pharmaceutics. 2024 Feb 1;16(2):215. doi: 10.3390/pharmaceutics16020215 (PMC10891968; doi:10.3390/pharmaceutics16020215)
Supplement: Supplementary file 1 [file pharmaceutics-16-00215-s001.zip › pharmaceutics-2841396-supplementary.pdf]

pharmaceutics

Supplementary Materials: The Table S1: Primers used in PCR; Figure S1: PCR amplification products obtained with 4 types of primers.

Table S1: Primers used in PCR.

Genes	Primers	Sequence	Amplicon size (bp)
gyrA	Sm_gyrA_F	CCAAGAATCTGCTGTCCG	111
	Sm_gyrA_R	TTGCGACTATCTGCTATGTG	
atpD	Sm_atpD_F	TGTTGATGGTCTGGGTGAAA	176
	Sm_atpD_R	TTTGACGGTCTCCGATAACC	
eno	Sm_eno_F	CAGCGTCTTCAGTTCCATCA	194
	Sm_eno_R	TCACTCAGATGCTCCAATCG	
lacG	Sm_lacG_F	ATTGGATGCGTGCTTTTGATGG	94
	Sm_lacG_R	CGACCGACACCCTTAATCTGG	
lacC	Sm_lacC_F	GCTGGAATTACATCGGCTCTTGC	157
	Sm_lacC_R	CCTCCGCTACCTCAATTTGTTGG	
ACT1	Ca_ACT1_F	TGCTCCAGAAGAACACCCA	182
	Ca_ACT1_R	CACCTGAATCCAAAACAATACCAGT	
HWP1	Ca_HWP1_F	TGGTGCTATTACTATTCCGG	182
	Ca_HWP1_R	CAATAATAGCAGCACCGAAG	
ECE1	Ca_ECE1_F	GCTGGTATCATTGCTGATAT	168
	Ca_ECE1_R	TTCGATGGATTGTTGAACAC	
CHT2	Ca_CHT2_F	TTGGGATGCTTCTGGGGCTT	111
	Ca_CHT2_R	GCAGAAGAAGATGGGGCAACAC	
ERG4	Ca_ERG4_F	TCAAATGTGCCAATGGTTCT	101
	Ca_ERG4_R	AGCCCAAGTCAATGTTTGAA	

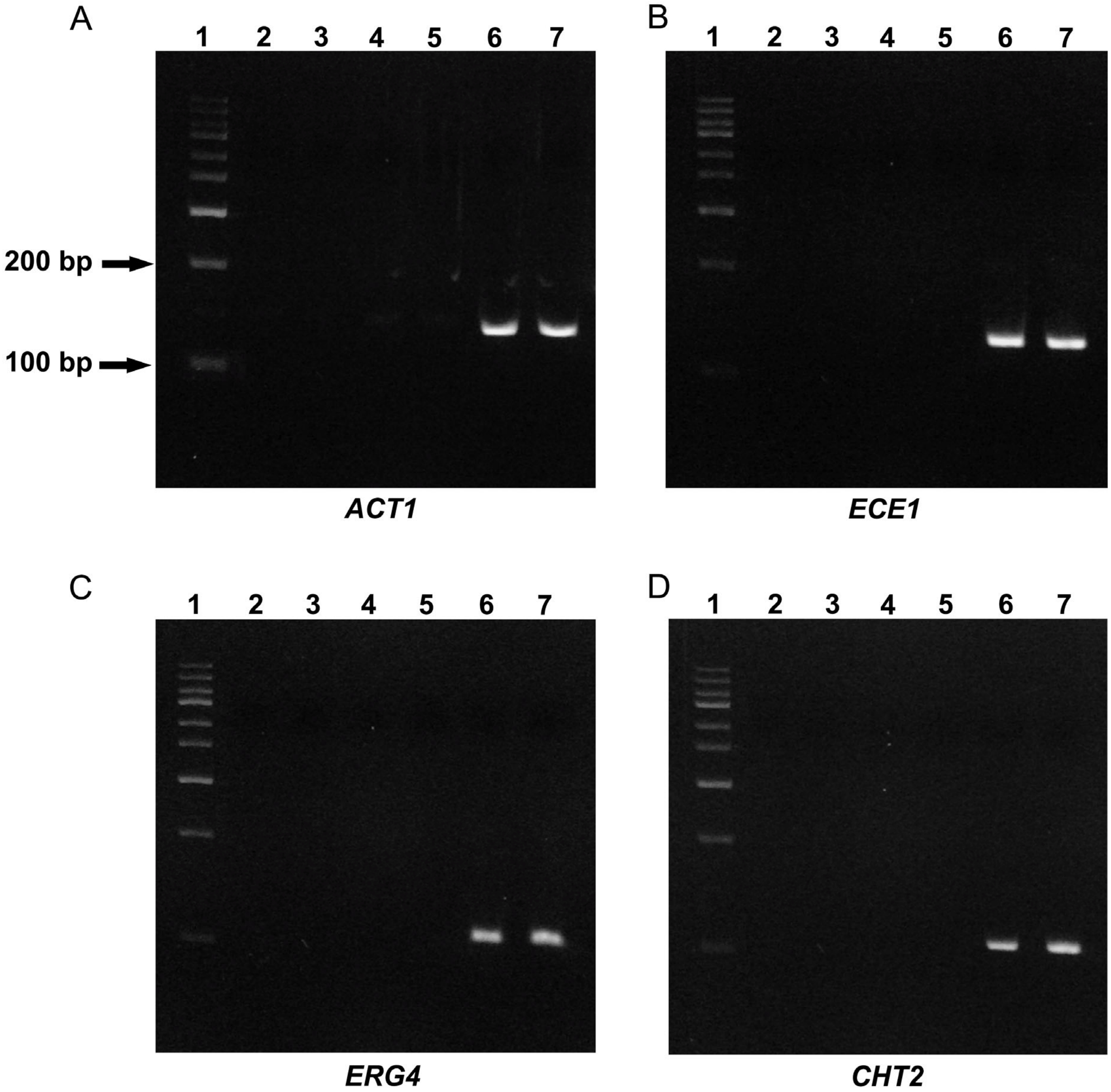

Figure S1: PCR amplification products obtained with 4 types of primers: *ACT1* (A), *ECE1* (B), *ERG4* (C), and *CHT2* (D). Lane 1 contains a 1 kb Plus DNA Ladder. Lane 2&3: *S. cerevisiae*; lane 4& 5: *S. boulardii*. Lane 6&7 shows amplification products from *C. albicans*.
